# Genome constitution and evolution of *Elytrigia lolioides* inferred from *Acc*1, *EF-G, ITS*, *TrnL-F* sequences and GISH

**DOI:** 10.1186/s12870-019-1779-x

**Published:** 2019-04-25

**Authors:** Long Wang, Yuanyuan Jiang, Qinghua Shi, Yi Wang, Lina Sha, Xing Fan, Houyang Kang, Haiqin Zhang, Genlou Sun, Li Zhang, Yonghong Zhou

**Affiliations:** 1 Triticeae Research Institute, Sichuan Agricultural University, Wenjiang, Chengdu, 611130 Sichuan China; 2 Key Laboratory of Crop Genetic Resources and Improvement, Ministry of Education, Sichuan Agricultural University, Wenjiang, Chengdu, 611130 Sichuan China; 3 College of Science, Sichuan Agricultural University, Ya’an, 625014 Sichuan China; 4 State Key Laboratory of Plant Cell and Chromosome Engineering, Institute of Genetics and Developmental Biology, Chinese Academy of Science, Beijing, 100101 China; 5 Biology Department, Saint Mary’s University, Halifax, Nova Scotia Canada

**Keywords:** *Elytrigia lolioides*, Genome constitution, Taxonomy, *Acc1*, *EF-G*, *ITS*, *trnL-F*, GISH

## Abstract

**Background:**

*Elytrigia lolioides* (Kar. et Kir.) Nevski, which is a perennial, cross-pollinating wheatgrass that is distributed in Russia and Kazakhstan, is classified into *Elytrigia*, *Elymus*, and *Lophopyrum* genera by taxonomists on the basis of different taxonomic classification systems. However, the genomic constitution of *E. lolioides* is still unknown. To identify the genome constitution and evolution of *E. lolioides*, we used single-copy nuclear genes acetyl-CoA carboxylase (*Acc1*) and elongation factor G *(EF-G*), multi-copy nuclear gene internal transcribed space (*ITS*), chloroplast gene *trnL-F* together with fluorescence and genomic in situ hybridization.

**Results:**

Despite the widespread homogenization of *ITS* sequences, two distinct lineages (genera *Pseudoroegneria* and *Hordeum*) were identified. *Acc1* and *EF-G* sequences suggested that in addition to *Pseudoroegneria* and *Hordeum*, unknown genome was the third potential donor of *E*. *lolioides*. Data from chloroplast DNA showed that *Pseudoroegneria* is the maternal donor of *E*. *lolioides*. Data from specific FISH marker for St genome indicated that *E*. *lolioides* has two sets of St genomes. Both genomic in situ hybridization (GISH) and fluorescence in situ hybridization (FISH) results confirmed the presence of *Hordeum* genome in this species. When E genome was used as the probe, no signal was found in 42 chromosomes. The E-like copy of *Acc1* sequences was detected in *E*. *lolioides* possibly due to the introgression from E genome species. One of the H chromosomes in the accession W6–26586 from Kazakhstan did not hybridize H genome signals but had St genome signals on the pericentromeric regions in the two-color GISH.

**Conclusions:**

Phylogenetic and in situ hybridization indicated the presence of two sets of *Pseudoroegneria* and one set of *Hordeum* genome in *E*. *lolioides*. The genome formula of *E*. *lolioides* was designed as StStStStHH. *E. lolioides* may have originated through the hybridization between tetraploid *Elymus* (StH) and diploid *Pseudoroegneria* species. E and unknown genomes may participate in the speciation of *E*. *lolioides* through introgression. According to the genome classification system, *E*. *lolioides* should be transferred into *Elymus* L. and renamed as *Elymus lolioidus* (Kar. er Kir.) Meld.

**Electronic supplementary material:**

The online version of this article (10.1186/s12870-019-1779-x) contains supplementary material, which is available to authorized users.

## Background

The taxonomic history of Triticeae mainly includes three stages, that is, artificial classification, natural or phonetic classification, and phylogenetic classification [1, 2]. Löve [3] divided Triticeae species into 37 genera according to the genomic system of classification, in which different species with the same genome or genome constitution were classified into one genus, although the justifiability of some genera remains controversial up to now. Many Triticeae species were reclassified into different genera on the basis of their genome constitutions [4–7]. However, the genome constitutions of many species with high ploidy in the genera *Elytrigia*, *Elymus*, and *Roegneria* in Triticeae still remain unknown or controversial.

*Elytrigia lolioides* (Kar. et Kir.) Nevski is a perennial, cross-pollinating wheatgrass that is distributed in Russia and Kazakhstan, has strong rhizomatous, and generally grows in stony mountain slopes, sandy land, and steppe [3, 8]. According to its morphological characteristics, *E*. *lolioides* is classified into different genera, including *Triticum*, *Agropyron*, *Elytrigia*, and *Elymus*, according to different classification systems [8, 9]. Cytological studies indicated that the chromosome number of *E*. *lolioides* is either 42 or 58 [10, 11]. It was suggested that this species contains St, E, and J genomes that are derived from *Pseudoroegneria* and *Lophopyrum elongatum* and *L. bessarabicum*, respectively, and is classified into *Elytrigia* [3]. Dewey [9] supported this treatment but indicated that *E*. *lolioides* has St and unknown genomes, and the genome formula was designed as StX (X is undetermined genome). Yen and Yang [8] speculated that *E*. *lolioides* should be classified into genus *Lophopyrum* with E genome. Tao and Lin [12] suggested that *E*. *lolioides* contains StE genome. Therefore, the genome constitution and origin of *E*. *lolioides* remain controversial. According to the Dewey taxonomic principle, *Elytrigia* genus has five species, including *E. repens*, *E. lolioides*, *E. pycnantha*, *E. pungens*, and *E. elongatiformis* [9]. Genome constitution of *E. repens*、*E. pycnantha*, and *E. pungens* were reported and these three species were classified into other genera [8]. Therefore, investigating genome constitution of *E. lolioides* will be useful to investigate the taxonomic status of *Elytrigia*.

Chromosome pairing at meiosis in artificial hybrid is commonly used to detect the genome constitution of species [13–16]. Chromosome pairing at the metaphase I of meiosis is convincing step in determining the genome constitution of the target species. However, interpreting chromosome pairing at high ploidy levels is difficult because of the difficulty in distinguishing autosyndetic and homoeologous pairing in meiosis [17]. Therefore, genomic in situ hybridization (GISH), specific molecular marker for genome or chromosome and phylogenetic analysis were used to investigate the genome constitution and origin of target species [18–21]. GISH is a fast and valuable tool to detect genome constitution and chromosomal translocation in species [22, 23]. Some genome-specific molecular and FISH markers were developed. These markers are stable and visual in detecting genome constitution and tracing target chromatin in wheat breeding program [24–26]. Phylogenetic analyses can identify the genome donors and introgression of polyploid. Single- or low-copy nuclear gene, multi-copy nuclear gene and cytoplasm gene have been successfully used to investigate parental and maternal origins [27–29]. Although undergoing concerted evolution, internal transcribed space (*ITS*) is a useful marker to conclude the genome origin of polyploid [30]. Acetyl-CoA carboxylase (*Acc1*) gene, elongation factor G (*EF-G*), *ITS*, and the space between tRNA-Leu and tRNA-Phe gene (*trnL-trnF* region) sequences have been used to examine the phylogenetic relationship, hybridization events, parental donor, and maternal origin [31–35].

In the present study, the specific molecular markers for St genome, GISH, single-copy nuclear genes *Acc1* and *EF-G*, multi-copy nuclear gene *ITS*, and chloroplast DNA *trnL-F* were used to investigate the genome constitution of *E*. *lolioides*. The objectives are as follows: (1) to detect the genome constitution and taxonomic treatment of *E. lolioides*, (2) to identify the maternal donor of *E*. *lolioides*, and (3) to clarify the origin of *E*. *lolioides*.

## Results

### Phylogenetic analyses of *ITS* sequence

**Fig. 1:**
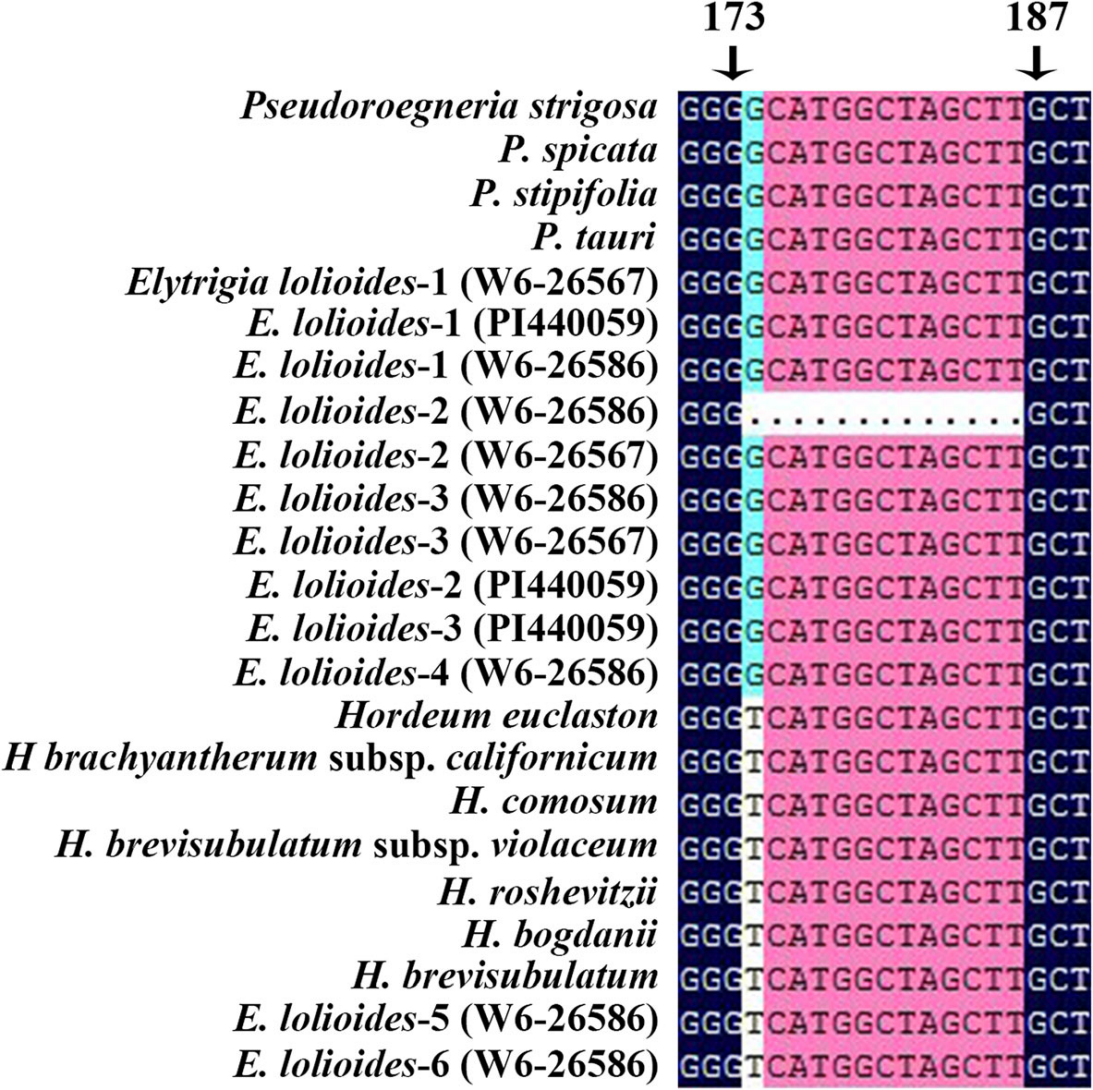
The deletion in *ITS1* region from *Elytrigia lolioides.* The 13 bp deletion from 176 to 188 bp in *ITS1* region of one *ITS* sequence from *Elytrigia lolioides* (W6–26586)

**Fig. 2:**
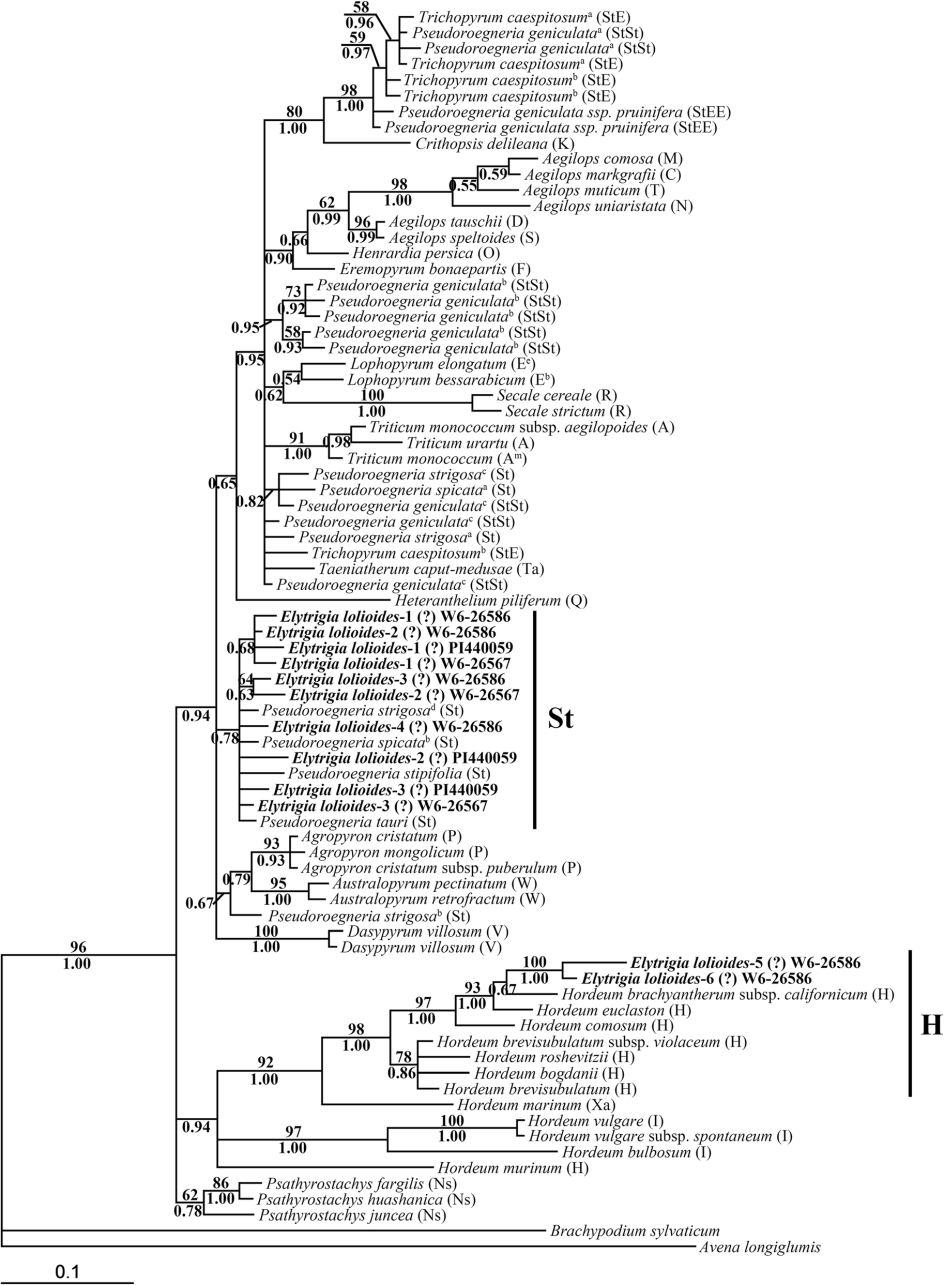
Strict consensus tree generated from *ITS* sequence data. Numbers above the branches were bootstrap support (BS) values and below the branches were Bayesian posterior probability (PP) values. The bold indicated sequences from three accessions of *Elytrigia lolioides*. The same superscript indicated the sequences from same accession

The length of *E*. *lolioides ITS* sequences ranged from 588 bp to 602 bp, and that of most sequences was ~ 601 bp. After multiple sequence alignments, the 13 bp deletion from 174 to 186 bp in the *ITS1* region was found in an *ITS* sequence from *E*. *lolioides* (W6–26586, Fig. 1). The *ITS* data matrix of 79 sequences was analyzed based on maximum likelihood (ML) by using the GTR + I + G model (−Ln likelihood = 4280.8823). *Brachypodium sylvaticum* and *Avena longiglumis* were used as the outgroup. A total of 626 characters were used for phylogenetic analysis, in which 351 were constants, 112 were parsimony uninformative, and 163 were parsimony informative. Finally, a single phylogenetic tree was yielded with the following assumed nucleotide frequencies: A = 0.22610, C = 0.28870, G = 0.27680, T = 0.20840. The trees generated by Bayesian analysis and ML were similar to each other. The ML tree with bootstrap support (BS) values (above the branch) and Bayesian posterior probability (PP, below the branch) is displayed in Fig. 2.

The multiple copies of sequences from each *E*. *lolioides* accession were separated into two distinct clades. One clade contained 10 sequences each from *E*. *lolioides* and diploid *Pseudoroegneria* species (PP = 0.78). The other clade contained two sequences each from *E*. *lolioides* and diploid *Hordeum* species (BS = 98%, PP = 1.00).

### Phylogenetic analyses of *Acc1* sequence

**Fig. 3:**
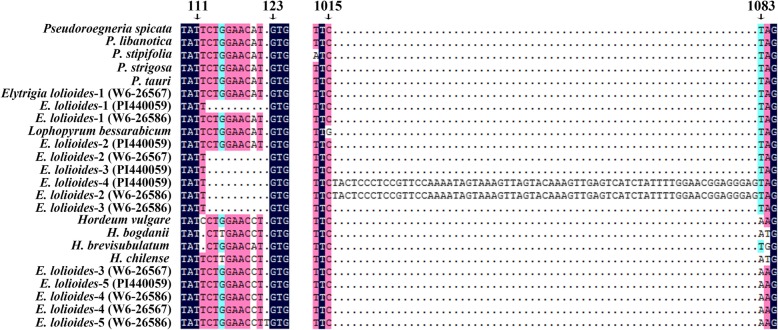
The deletion and insertion in *Acc1* sequnces from *Elytrigia lolioides.* The 10 bp deletion from 110 to 119 bp and 67 bp insertion from 1015 to 1081 bp were found in intron 1 and intron 5 regions of *Acc1* sequences from *Elytrigia lolioides*, respectively

The 31 and 38 positive clones were sequenced for the three accessions of *E*. *lolioides*. The length of these sequences ranged from 1400 bp to 1495 bp, and that of most sequences was ~ 1440 bp. All sequences contained eight exons and seven introns. These findings are similar to the results in a previous study [36]. After multiple sequence alignments, 10 bp deletion was found in 43 sequences (17/31 in PI 440059, 14/38 in W6–26586, 12/28 in W6–26567) from the 110–119 position in intron 1 region. A 67 bp insertion at the 1015–1081 position in intron 5 region was found in two sequences from the two accessions of *E*. *lolioides* (PI440059 and W6–26586, Fig. 3). Blast search indicated that the 67 bp insertion belongs to Tc1 DNA transposon. Termination codon was found in the exon 5 of the two sequences from accession W6–26586, and these sequences were excluded in the phylogenetic analysis.

**Fig. 4:**
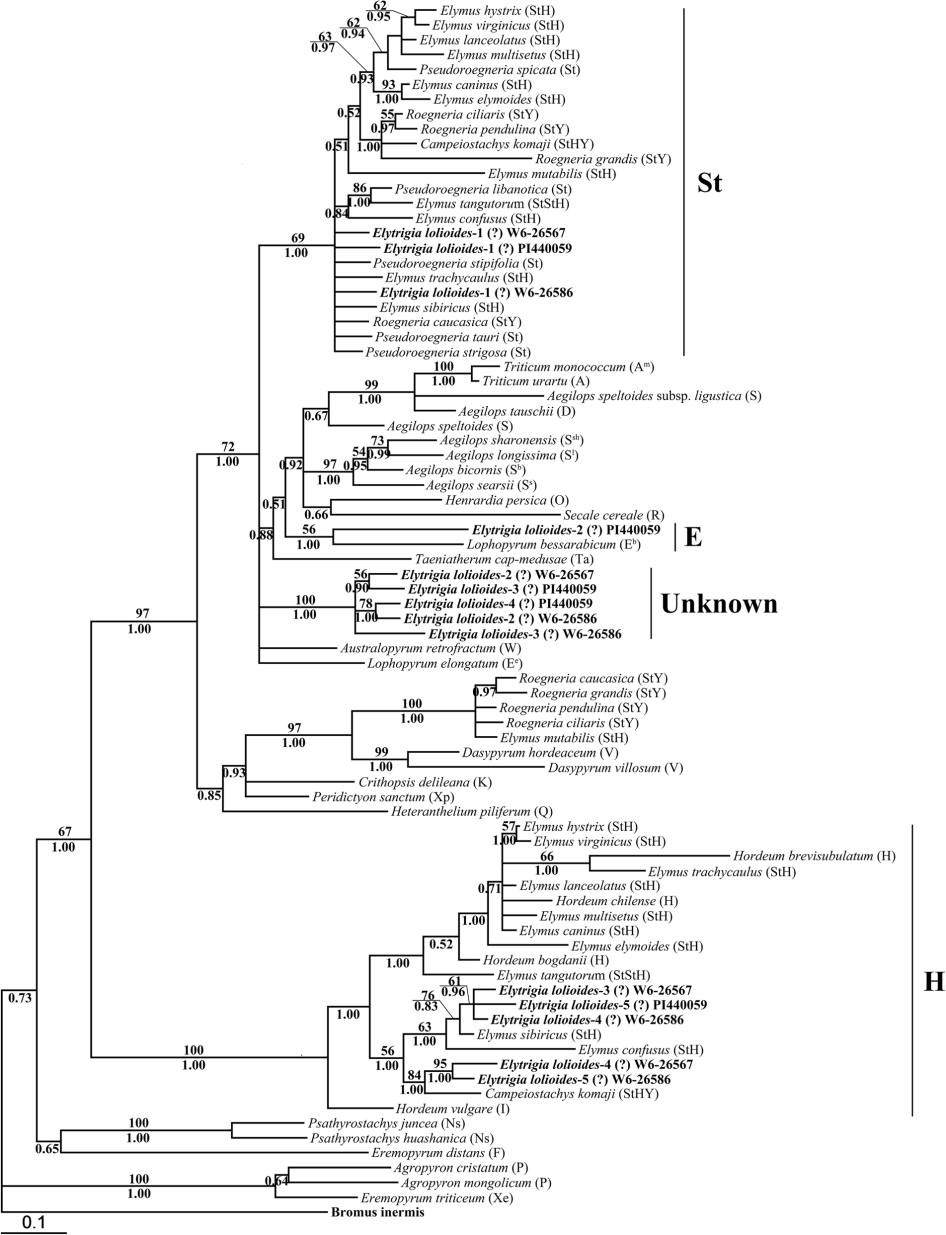
Strict consensus tree generated from *Acc1* sequence data. Numbers above the branches were bootstrap support values (BS) and below the branches were Bayesian posterior probability (PP) values. The bold indicated sequences from three accessions of *Elytrigia lolioides*

The phylogenetic analysis of the 82 *Acc*1 sequences was performed using *Bromus inermis* as the outgroup. The data matrix contained 1885 characters, 1353 of which were constants, 257 were parsimony uninformative, and 275 were parsimony informative. A signal phylogenetic tree generating by maximum likelihood analysis using the GTR + I + G model (−Ln likelihood = 8780.6729; assumed nucleotide frequencies: A = 0.25250, C = 0.18430, G = 0.21920, T = 0.34400) is shown in Fig. 4 with the BS from ML and PP values from Bayesian analyses.

Three distinct *Acc1* copies of the sequences from each three accessions of *E*. *lolioides* were grouped into three different clades. The first clade was St clade, which included five diploid *Pseudoroegneria* species, tetraploid *Elymus* and *Roegneria* species, hexaploid species of *Campeiostachys* and *E*. *lolioides* (BS = 69%, PP = 1.00). In the St clade, *Pseudoroegneria tauri*, *P. strigosa*, *P*. *stipifolia*, *Elymus sibiricus*, *Ely. trachycaulus*, *Roegneria caucasica*, and three *E*. *lolioides* formed a paraphyletic grade. The second clade was the H genome clade, which contained the diploid species of *Hordeum*, tetraploid species of *Elymus*, hexaploid species of *Campeiostachys* and *E*. *lolioides* (BS = 100%, PP = 1.00). In the second clade, five *E*. *lolioides* sequences, two tetraploid species of *Elymus* (*Ely. sibiricus* and *Ely. confuses*) and *Campeiostachys kamoji* formed a subclade (PP = 56%, BS = 1.00). However, the third clade only contained sequences from *E*. *lolioides* without any diploid species (BS = 100%, PP = 1.00). One sequence each from the accession PI440059 of *E. lolioides* and *L*. *bessarabicum* was grouped together (BS = 56%, PP = 1.00). The *Acc1* data displayed an evident Y genome clade (BS = 100%, PP = 1.00), and no sequences from *E*. *lolioides* were grouped in this clade.

### Phylogenetic analysis of *EF-G* sequence

**Fig. 5:**
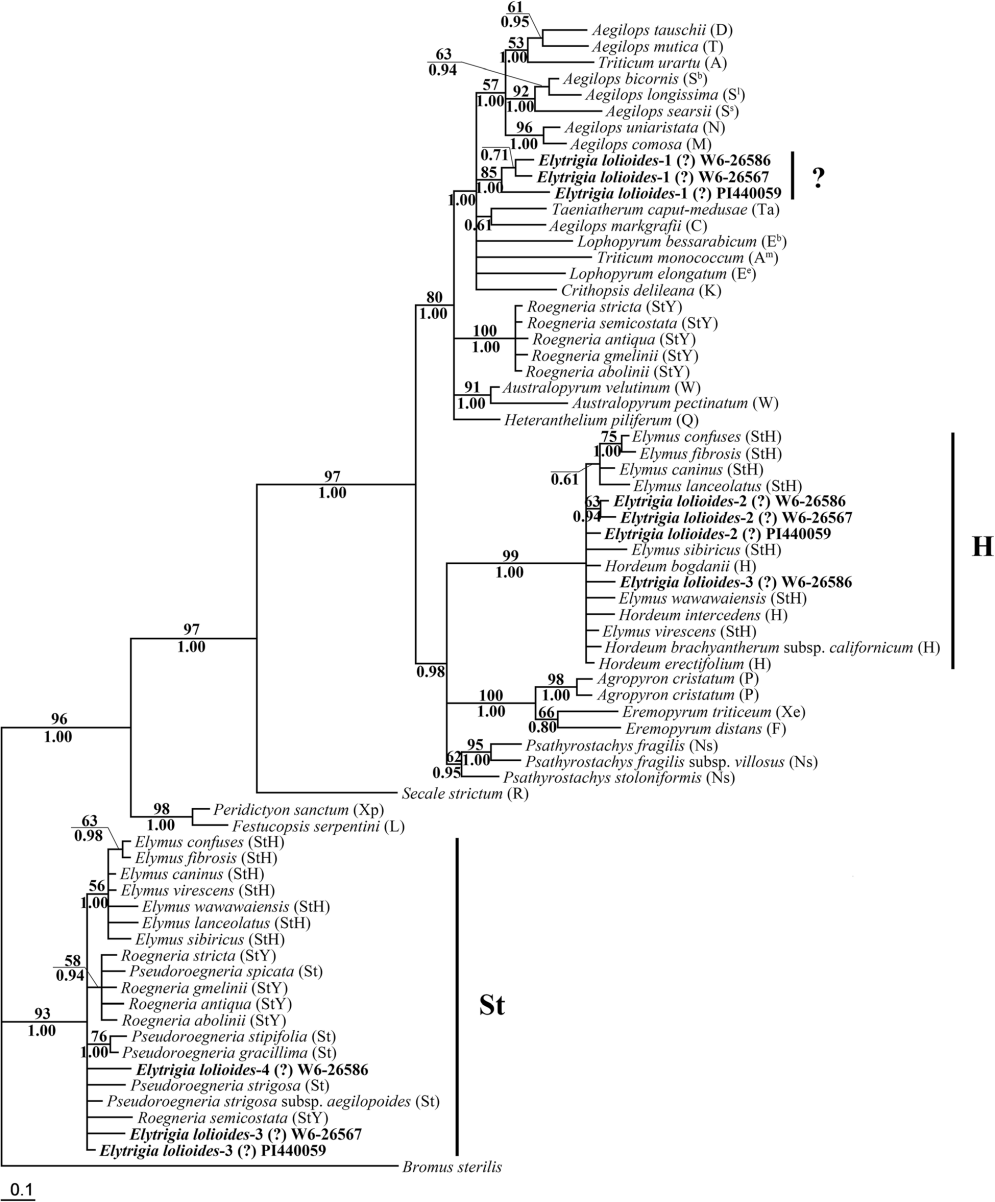
Strict consensus tree generated from *EF-G* sequence data. Numbers above the branches were bootstrap support values (BS) and below the branches were Bayesian posterior probability (PP) values. The bold indicated sequences from three accessions of *Elytrigia lolioides*

The *EF-G* matrix contained 71 taxa and 870 characters, 590 of which were constants, 127 were parsimony uninformative, and 153 were parsimony informative. HKY + G, as the best-fit model (−Ln likelihood = 3898.0823), was used in phylogenetic analysis. A single phylogenetic tree was yielded and the assumed nucleotide frequencies: A = 0.26340, C = 0.19760, G = 0.21780, T = 0.32120. The tree generated by Bayesian analyses and ML were similar to each other. The ML tree with BS values (above the branch) and Bayesian PP (below the branch) is displayed in Fig. 5.

**Fig. 6:**
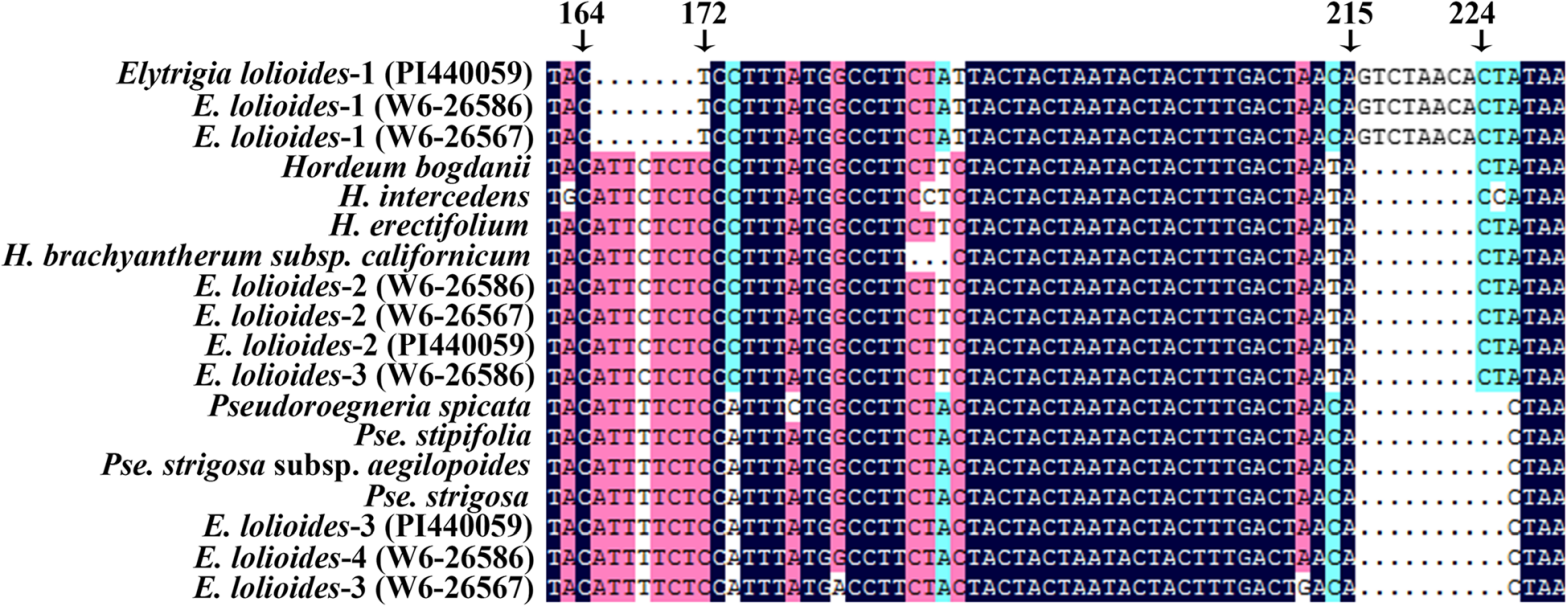
The deletion and insertion in *EF-G* sequnces from *Elytrigia lolioides.* The 7 bp deletion and 8 bp insertion in position 164–172 bp and 215–224 bp were found in EF-G sequences from Elytrigia lolioides, respectively

The phylogenetic analyses of the *EF-G* sequence distinctly separated the three copies of sequences from the three accessions of *E*. *lolioides* into three different clades. The first clade was St clade, which included five *Pseudoroegneria* species, seven tetraploid *Elymus* species, five tetraploid *Roegneria* species and *E*. *lolioides* (PP = 93%, BS = 1.00). The second clade was the H clade, which contained the diploid of *Hordeum* species, seven tetraploid *Elymus* species and *E*. *lolioides* (PP = 99%, BS = 1.00). However, in the third clade, all sequences were only from *E*. *lolioides* that were grouped together (PP = 85%, BS = 1.00) and sisters to the sequences from diploid *Aegilops*, *Triticum*, *Lophopyrum*, *Taeniatherum*, and *Crithopsis* species. All these sequences had 7 bp deletion and 8 bp insertion in 164–172 and 215–224 bp positions, respectively (Fig. 6). Meanwhile, *EF-G* data displayed an evident Y genome clade (BS = 100%, PP = 1.00) and without any sequence from *E*. *lolioides*, which is similar to the results of the *Acc1* phylogenetic tree.

### Phylogenetic analyses of *trnL-F* sequence

**Fig. 7:**
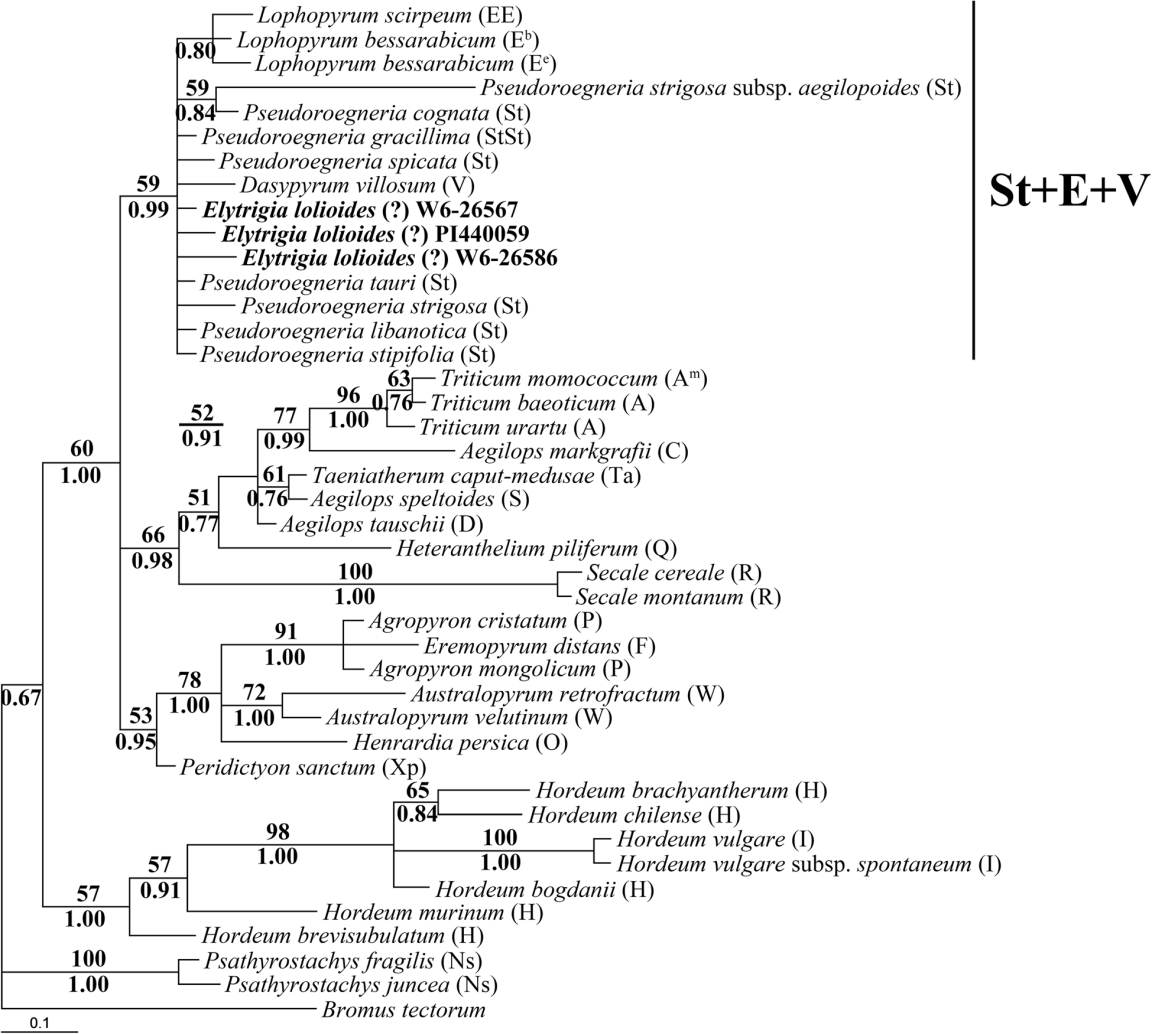
Strict consensus tree generated from *trnL-F* sequence data. Numbers above the branches are bootstrap support values and below the branches were Bayesian posterior probability (PP) values. The bold indicated sequences from three accessions of *Elytrigia lolioides*

A total of 42 *trnL-F* sequences were selected for ML analysis. *B. tectorum* was used as the outgroup. The data matrix contained 1013 characters, 884 of which were constants, 56 were parsimony uninformative, and 73 were parsimony informative. HKY + G as the best-fit model (−Ln likelihood = 2435.1624) was used in phylogenetic analysis, and a single phylogenetic tree was yielded. The assumed nucleotide frequencies were A = 0.33724, C = 0.15580, G = 0.13259, T = 0.37437. The tree generated by Bayesian analyses was similar to ML tree. The ML tree with BS values (above the branch) and Bayesian PP (below the branch) is shown in Fig. 7.

All *trnL-F* sequences were distinctly divided into five clades. The sequences from *E*. *lolioides* were grouped with diploid *Pseudoroegneria*, *Lophopyrum*, and *Dasypyrum* species (BS = 59%, PP = 0.99). In this clade, sequences from *Lophopyrum* species which contained E genome, formed a subclade (PP = 0.80).

### In situ hybridization

**Fig. 8:**
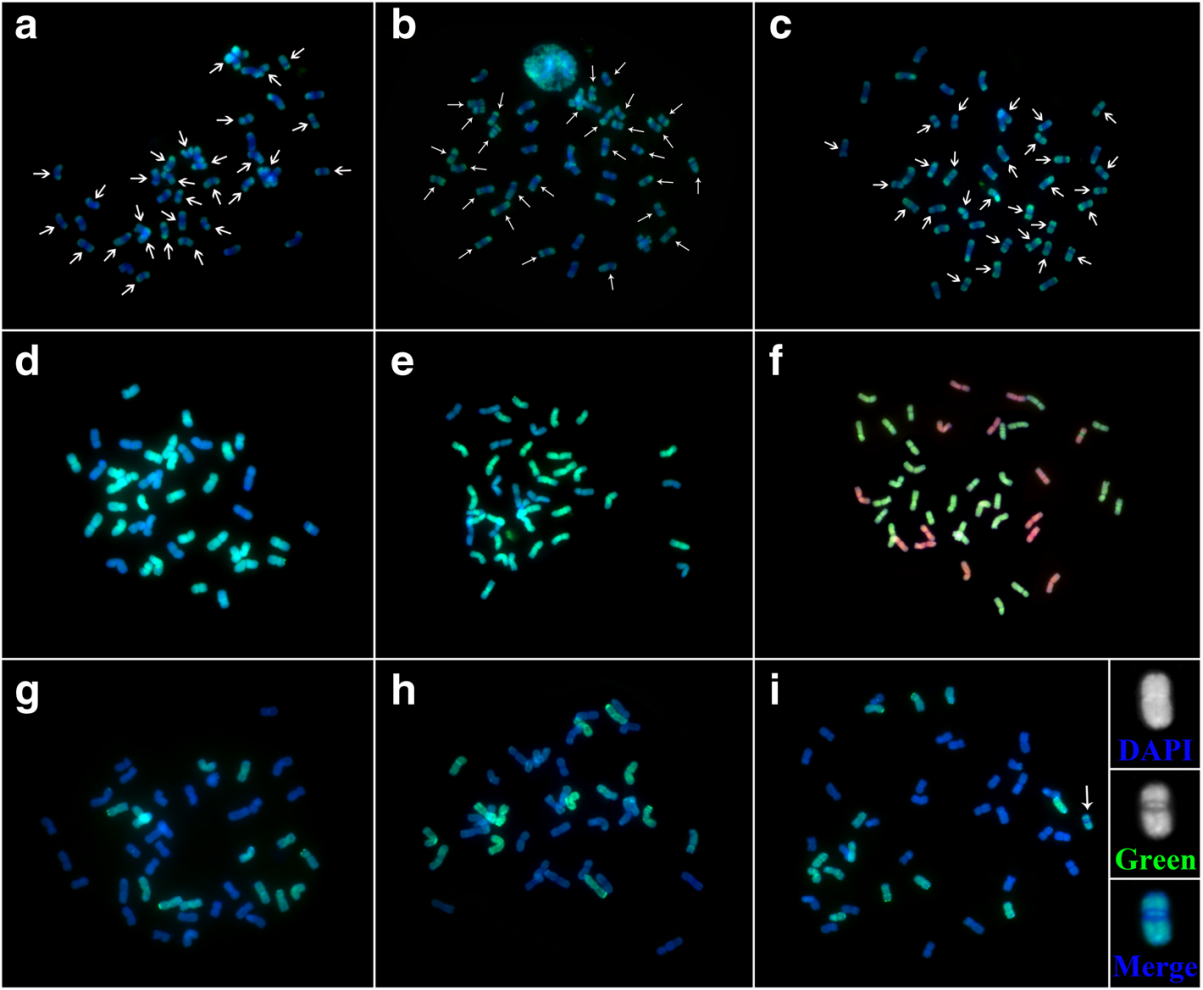
Results of FISH and GISH in *Elytrigia lolioides.* **a** and **g**: PI 440059, **b** and **h**: W6–26567, **c**-**f** and **i**: W6–26586. **a**-**c**: Used St_2_–80 as probe, 28 chromosomes were labeled as St type (arrows). The rest of the chromosomes were labeled as non-St type in three accessions of *E. lolioides*, respectively. **d**: Total genomic DNA of *Pseudoroegneria libanotica* was labeled with fluorescein-12-dUTP (green) as probe, 28 chromosomes were hybridized with *P. libanotica* probe (St genome). **e**: Total genomic DNA of *P. libanotica* labeled with fluorescein-12-dUTP (green) and total genomic DNA of *Lophopyrum bessarabicum* labeled with Texas-red-5-dCTP (red) as probes, 28 chromosomes were hybridized with the *P. libanotica* probe (**St** genome) and no any E genome signals were displayed on the chromosomes. **f**: Total genomic DNA of *P. libanotica* labeled with fluorescein-12-dUTP (green) and total genomic DNA of *Hordeum bogdanii* labeled with Texas-red-5-dCTP (red) as probes, 28 chromosomes were hybridized with the *P. libanotica* probe (**St** genome) and the rest were hybridized with the *H. bogdanii* probe (**H** genome). **g**-**i**: Total genomic DNA of *H. bogdanii* labeled with fluorescein-12-dUTP (green), 14 chromosomes were hybridized with the *H. bogdanii* probe (**H** genome) in three accessions of *E. lolioides*, respectively. However, in accession of W6–26586, one chromosome which belongs to H genome did not hybridized H genome signals on the pericentromeric regions (arrow). Insets showed the magnified view of the signal on this chromosome

Chromosome number analysis results indicated that *E*. *lolioides* is a hexaploid (2n = 6x = 42) wheatgrass. St_2_–80 is a FISH marker for St genome. Signals produced by St_2_–80 were present on the entire arm of the St genome chromosomes, except at the centromeric region. However, signals produced by St_2_–80 were present in the terminal region of the E and H genome chromosomes [26]. This marker was used to detect St genome in the *E*. *lolioides* chromosomes. The signal on 28 chromosomes was displayed St type (Fig. 8a, b, and c). This result was confirmed by GISH, wherein 28 chromosomes were hybridized with the St probe from *Pseudoroegneria libanotica* (Fig. 8d). To detect the 14 other chromosomes, E (from *Lophopyrum bessarabicum*) and H (from *Hordeum bogdanii*) genomes were used as probes. After two-color GISH, no any E genome signals were displayed on the chromosomes (Fig. 8e). However, the chromosomes that did not hybridize with St genome displayed intense signals when probed by the H genome (Fig. 8f). Then, the test was performed using H genomes as probes in the three *E*. *lolioides* accessions (Fig. 8g, h, and i). Minor disparity was displayed in chromosome of W6–26586 that one chromosome which belongs to H genome did not hybridized H genome signals on the pericentromeric regions (Fig. 8i).

## Discussion

### Maternal donor of *E. lolioides*

The chloroplast DNA (cpDNA) is maternally inherited in grasses [37]. Several sequences, including space and coding regions (e.g., *trnL-F*, *trnD-T*, *trnH-psbA*, *matK*, *Rps16*, and *rbcL*) were used to identify the maternal donor of wheatgrass or genera in Triticeae [29, 32, 34, 38, 39]. In the present study, all sequences from *E*. *lolioides* with the sequences from the diploid *Pseudoroegneria* (St) and *Dasypyrum* (V) and the diploid and tetraploid *Lophopyrum* (E) formed a clade (BS = 59%, PP = 0.99) in *trnL-F* data. It seems to suggest that *Pseudoroegneria*, *Dasypyrum*, and *Lophopyrum* were the potential maternal donors of *E*. *lolioides*. In a previous study, close relationships among *Pseudoroegneria*, *Dasypyrum*, and *Lophopyrum* were discovered using the cpDNA data from diploid species in Triticeae [33, 40]. In contrast to the *trnL*-*F* data, *Acc1* sequences from *Pseudoroegneria*, *Dasypyrum*, *Lophopyrum*, *Hordeum*, and other diploids were distinctly separated into different clades, and sequences from *E*. *lolioides* were placed into *Pseudoroegneria*, *Lophopyrum*, and *Hordeum* clades. No chromosomes that were hybridized with E probe were derived from *L*. *bessarabicum* in GISH. Thus, we infer *Pseudoroegneria* is a maternal donor of *E*. *lolioides*. Numerous studies indicated that *Pseudoroegneria* is a maternal donor of polyploid species containing St genome in Triticeae [41–44]. The female species in Triticeae carrying St genome are successful in terms of distant hybridization [45].

### Origin of *E*. *lolioides*

Our results indicated that *Pseudoroegneria* is the major genome donor for *E*. *lolioides*. GISH and FISH indicated that *E*. *lolioides* had two sets of St genome. In strict consensus tree generated from the *Acc1* sequence, several *Acc1* sequences from the three accessions of *E. lolioides* formed a clade without any sequence from the diploid species in Triticeae. Meanwhile, all of these sequences had a 10 bp deletion that is absent in the other published *Acc1* sequences of Triticeae species from the 110 to 119 positions in intron 1 region. Some non-synonymous substitutions were discovered in several of these sequences. One sequence that was placed into the St clade also had this deletion. The preliminary results in our laboratory also showed that the same deletion was found in *Acc1* sequences from *E. pungens*, *Psammopyrum athericum*, and *E*. *elongatiformis*, which were collected from the Middle East (unpublished). Two of these sequences had 67 bp insertions at the 1015–1081 bp positions in intron 5. And in strict consensus tree generated from the *EF-G* sequence, three sequences from the three accessions of *E*. *lolioides* that formed a clade without other sequences were observed. Similarly as *Acc1* sequences, these sequences also contained special insert fragments. Therefore, sequences with special indel may be derived from an independent diploid species, which is extinct or unknown. This hypothesis will be validated by checking whether *Acc1* and *EF-G* sequences with special indel were obtained from Triticeae diploid species in the Middle East.

The contribution from *Hordeum* to the accessions of *E*. *lolioides* was indicated by the data of *Acc1*, *EF-G*, and *ITS* sequences with high support and also confirmed by GISH. However, the *Hordeum*-like copy of *ITS* sequence was not obtained from accessions PI 440059 and W6–26567 possibly due to concerted evolution. In the *Acc1* data, *E. lolioides* and *Elymus* species (StH) formed a subclade without the diploid *Hordeum* species in H clade. It can be concluded that the tetraploid *Elymus* species was the direct donor during *E*. *lolioides* speciation. One chromosome had intensive hybridization signals derived from *H*. *bogdanii* on the arms but had clear signals derived from *P*. *libanotica* on the pericentromeric regions in GISH. Previous studies showed that these mosaic chromosomes are observed in *Psammopyrum athericum* (StEP/LEP) [46]*, E. pungens* (StStEP/StLEP) [47], *Elymus repens* (StStH) [19], *Thinopyrum intermedium* (StEE) [20], and *T. ponticum* (StStEEE/EEEEE) [48] by genomic in situ hybridization. Thus, the chromosome rearrangement between St and the other genomes (i.e., E, H, and P) or the retrotransposon activity of St genome may lead to mosaic chromosomes after allopolyploidization. Interestingly, most of these mosaic chromosomes have the same character that St genome signal appeared on the centromeric or pericentromeric region of the other genomes. Redinbaugh et al. [45] found that the female species carrying the St genome are more successful in terms of distant hybridization. Those rearrangement or retrotransposon insertion may contribute to the stability of different genomes after allopolyploidization.

In the *Acc1* data, one sequence from *E*. *lolioides* (PI440059) and *L*. *bessarabicum* formed a clade with moderate support (BS = 56%, PP = 1.00). Tao and Lin [12] produced a specific SCAR marker, which was used to detect StE genome in Triticeae, thereby indicating that *E*. *lolioides* contain StE genomes. However, parental donor from E genome was undiscovered by *EF-G*, *ITS* data, and GISH. The *Lophopyrum*-like *Acc1* copy that was obtained from *E*. *lolioides* is likely caused by introgression [49]. *L*. *bessarabicum* is a diploid species that is distributed in Mediterranean Sea, Azov Sea, and Euxine Sea. Therefore, overlapping the geographical area between *L*. *bessarabicum* and *E*. *lolioides* can increase the possibility for introgression.

**Fig. 9:**
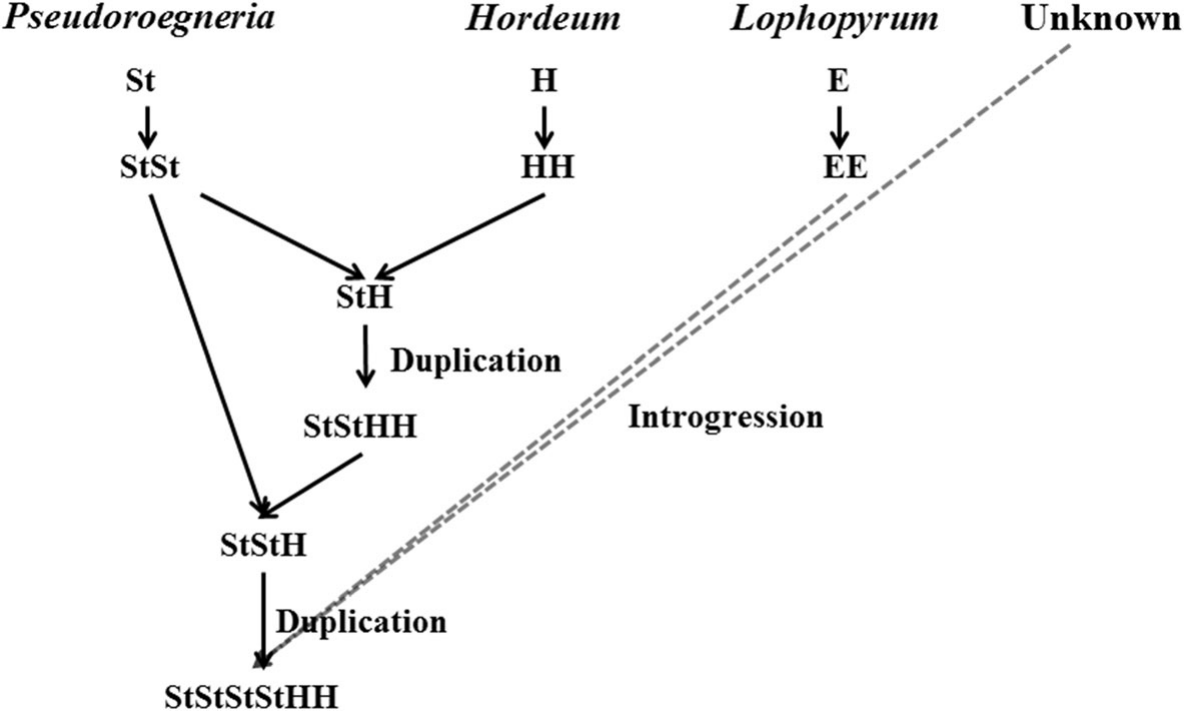
A hypothetical scenario about the origin of *Elytrigia lolioides* inferred from the three sequences data and in situ hybridization. Contribution of major genome constituents (*Pseudoroegneria* and *Hordeum*) were displayed with solid lines and hypothetical donor was displayed with dashed lines

The hypothetical scenarios of the origin of *E*. *lolioides* origin were suggested by combining the data of the four sequences and in situ hybridization. *E*. *lolioides* may have originated through the hybridization between tetraploid *Elymus* (StH) and diploid of *Pseudoroegneria* species, and then followed by whole genome duplication. After hybridization and polyploidy, the transposon or retrotransposon activation and E and unknown genome introgression may participate in *E*. *lolioides* speciation (Fig. 9).

### Genomic constitution of *E*. *lolioides*

*E*. *lolioides* is a perennial wheatgrass that was first reported by Karelin and Kiriloff in 1841. The chromosome number of *E. lolioides* is controversial because different ploidy levels were reported. Schulz-Schaeffer and Jurasits [11] reported that the accession PI 223325 of *Agropyron lolioides* (= *Elytrigia lolioides*) has 58 chromosomes. However, when this accession was rechecked in the US National Plant Germplasm System, it was named as *Elymus repens subsp. elongatiformis* (https://npgsweb.ars-grin.gov/gringlobal/accessiondetail.aspx?id=1180280), which is an octaploid grass and distributed in Central Asia and southern Russia [50]. Considering the report of Löve [10] and our present results, *E*. *lolioides* (2n = 42) is a hexaploid wheatgrass. In the present study, *Pseudoroegneria* and *Hordeum* were regarded as the major progenitors for *E*. *lolioides* as indicated by the data of the three nuclear gene (*Acc1*, *EF-G*, and *ITS*) sequences. Both GISH and FISH results indicated that *E*. *lolioides* had two sets of St genome and one set of H genome. However, in the strict consensus tree generated from *EF-G* and *Acc1* sequences, several sequences from the three accessions of *E*. *lolioides* formed a clade without any sequence from the diploid species in Triticeae. All these sequences had a special deletion or insertion that is not present in other published sequences for Triticeae species. These sequences may be contributed by unknown species, which were extinct or undiscovered. The contribution from *Lophopyrum* to *E. lolioides* was only indicated by the *Acc1* data with moderate support. However, parental donor from E genome was undiscovered by *EF-G*, *ITS* data, and GISH. The *Lophopyrum*-like *Acc1* copy that was obtained from *E*. *lolioides* was likely caused by introgression. Our results indicated that *E*. *lolioides* contained two sets of St genome and one set of H genome, and the genome formula was designed as StStStStHH.

### Classification status of *E. lolioides*

The main morphological characteristics of *E. lolioides* included long, creeping rhizome, long anther, apex subobtuse of lemmas, spikelet sessile, and one spikelet per node of rachis. The mid-nerve of lemma slightly extended and formed an extremely short point. According to the morphological characteristics, this species was successively classified into *Agropyron*, *Elytrigia*, and *Elymus* by an agrostologist with different classification systems [9]. The problem on the classification of *E*. *lolioides* was mainly caused by the taxonomic status of *Elytrigia* genus that was established by Desvaux in 1810, and then treated as a genus, sectional status within *Agropyron*, subgenus of *Agropyron* or section of *Elymus* by different taxonomists, respectively [8] According to genome constitution, *E*. *lolioides* was classified into *Elytrigia* genus that genome constitution of either SJE [3] or SX [9]. Yen and Yang [8] indicated that this species may be classified into *Lophopyrum* with E genome. Our results suggested that the genomic constitution of *E*. *lolioides* was StStStStHH. According to the genome classification system, this species should be transferred into *Elymus* L. and renamed as *Elymus lolioidus* (Kar. er Kir.) Meld..

## Conclusions

According to our study, *E. lolioides* is a hexaploid wheatgrass. *Pseudoroegneria* and *Hordeum* are major genome donors. E and unknown genomes may participate in the speciation of *E*. *lolioides* through introgression. This species should be transferred into *Elymus* L by combining four sequences data and in situ hybridization. The results of this study will help in investigating the taxonomic status of *Elytrigia* genus.

## Methods

### Plant materials and DNA extraction

The seeds of the three *E. lolioides* accessions from Former Soviet Union and Kazakhstan were provided by the Germplasm Resources Information Network of the United States Department of Agriculture (USDA). The total genomic DNA was extracted from fresh leaf tissues by using the CTAB method [51]. Three nuclear genes (*Acc1*, *EF-G*, and *ITS*) and one chloroplast *trnL-F* sequence from *E*. *lolioides* were amplified and sequenced. *Acc1*, *EF-G*, *ITS*, and *trnL-F* sequences from polyploid and diploid species representing A, S, D, E, W, St, V, K, Xp, Q, H, I, Ns, F, P, and Xe genomes in Triticeae were downloaded from GenBank (http://www.ncbi.nlm.nih.gov) and included in phylogenetic analysis. The basic information about these sequences, including genomic constitutions and GenBank identification numbers, are listed in Additional file 1: Table S1. The voucher specimens of *E. lolioides* were deposited in the Herbarium of Triticeae Research Institute, Sichuan Agricultural University, China.

### Amplification and sequencing

Low-copy and multi-copy nuclear genes (*Acc1*, *EF-G* and *ITS*) and the chloroplast gene *trnL-F* sequences were amplified via polymerase chain reaction (PCR) by using the primers of cMWG699T3–2 and CMWG699T7–2 [52], AccF1 and AccF2 [53], ITSL and ITS4 [54], and c and f [55], respectively. The primers and PCR profiles for the *Acc1*, *EF-G*, *ITS*, and *trnL-F* genes are listed in Table 1. Sequences were amplified in a 25 μL reaction mixture containing 50 ng template DNA, 1× reaction buffer, 2 mM MgCl_2_, 0.4 μM of each primer, 200 μM dNTP, and 1.5 U ExTaq. In addition, 8% dimethylsulfoxide (DMSO) was added to avoid influence of higher GC content during *ITS* sequence amplification [28]. The PCR products were detected on 1.0% agarose gels and then cloned into a PMD19-T vector according to the manufacturer’s instructions (TaKaRa, China). After white-blue plaque selection, 30–40 randomly selected clones of *Acc1*, *EF-G*, and *ITS* and 5 clones of *trnL-F* for each accession were sequenced in both directions by Shanghai Sangon Biological Engineering and Technology Service Ltd. (Shanghai, China).

**Table 1:** The primers and PCR condition for three genes

Gene	Name of primers	Sequences of primers (5′-3′)	Profiles
*Acc1*	Acc1F1	CCCAATATTTATCATGAGACTTGCA	1 cycle: 5 min 94 °C; 35 cycles: 30s 94 °C, 30s 56 °C, 2 min 30s 68 °C; 1 cycle 10 min 68 °C.
	Acc1F2	CAACATTTGAATGAAThCTCCACG	
*EF-G*	cMWG699T3–2	AACTGTTTTCTCATTTGTGA	1 cycle: 5 min 94 °C; 35 cycles: 30s 94 °C, 30s 55 °C, 1 min 30s 72 °C; 1 cycle 10 min 72 °C.
	cMWG699T7–2	AAGTGTCCTTGCCTTCCAAA	
*ITS*	ITSL	TCGTAACAAGGTTTCCGTAGGTG	1 cycle: 5 min 94 °C; 35 cycles: 30s 94 °C, 1 min 50 s 55 °C, 1 min 50 s 72 °C; 1 cycle 10 min 72 °C.
	ITS4	TCCTCCGCTTATTGATATGC	
*TrnL-F*	c	CGAAATCGGTAGACGCTACG	1 cycle: 5 min 94 °C; 35 cycles: 1 min 94 °C, 1 min 55 °C, 1 min 72 °C; 1 cycle 10 min 72 °C.
	f	ATTTGAACTGGTGACACGAG	

### Data analysis

Multiple sequence alignments were made using MAFFT 7.3 [56] and adjusted manually. The phylogenetic analyses of *Acc1*, *EF-G*, *ITS*, and *trnL-F* data by using the ML method was performed using PAUP*4.0bet10 (Swofford DL, Sinauer Associates, http://www.sinauer.com). The best-fit evolutionary model for phylogenetic analysis was determined using ModelTest v3.7 with Akaike information criterion [57]. ML heuristic searches were performed with 100 random addition sequence replications and Tree Bisection-Reconnection (TBR) branch swapping algorithm. As a measurement of the robustness of tree clades, the BS values were calculated with 1000 replications and displayed in figure (above the branch) if the BS values were > 50% [58].

In addition to ML analysis, Bayesian analyses were also performed using MrBayes 3.1 [59]. The evolutionary model selected for Bayesian analyses was same as ML analysis. Two sets of four chains were run 3.3 million generations for *Acc1* data, 4.2 million generations for *EF-G* data, 6.5 million generations for *ITS* data, and 0.54 million generations for *trnL-F* data, and samples were taken and saved every 100 generation under best-fit model. After discarding the first 25% samples as “burn-in”, a majority rule consensus tree with PP value (under the branch) was obtained.

### Chromosome preparation, fluorescence, and multicolor genomic in situ hybridization

Rapidly growing roots were collected from adult plants. The roots were treated with N_2_O for 2 h at 0.1 MPa and then fixed for 5 min with 90% glacial acetic acid. Chromosomes were prepared for analysis by using a previously reported method [60]. Plasmid DNA with St_2_–80 sequence was extracted using the EndoFree Plasmid Mini Kit (Tiangen, China), which was used to distinguish the chromosomes of St genome and others, including A, B, D, E, H, P, and Y genomes [26], according to the manufacturer’s instructions. The total genomic DNA of *P*. *libanotica* and plasmid (contained St_2_–80 sequence) were labeled with fluorescein-12-dUTP, and the genomic DNA of *L*. *bessarabicum* and *H*. *bogdanii* were labeled with Texas-red-5-dCTP by using the nick translation method. Hybridization procedure was performed according to the previously reported method [61]. Slides were detected under an Olympus BX53 fluorescence microscope with camera. At least five metaphase cells for each accession were analyzed. Adobe Photoshop was used to proceed the color images.

## Additional file

Additional file 1: **Table S1.** The related species in Triticeae used in this study. (XLSX 21 kb)

## Abbreviations

*Acc1*: Acetyl-CoA carboxylase 1
AIC: Akaike information criterion
BS: Bootstrap support
cpDNA: Chloroplast DNA
DMSO: Dimethylsulfoxide
EF-G: elongation factor G
FISH: Fluorescence in situ hybridization
GISH: Genomic in situ hybridization
ITS: Internal transcribed space
PCR: Polymerase chain reaction
PP: Posterior probability
rDNA: Ribosomal DNA
SCAR: Sequence characterized amplified region
TBR: Tree bisection-reconnection
*TrnL-F*: Intergenic spacer between transfer RNA gene trnL (UAA) and trnF (GAA)
